# Reliability of Motor Evoked Potentials Induced by Transcranial Magnetic Stimulation: The Effects of Initial Motor Evoked Potentials Removal

**DOI:** 10.15412/J.BCN.03080106

**Published:** 2017-01

**Authors:** Fahimeh Hashemirad, Maryam Zoghi, Paul B Fitzgerald, Shapour Jaberzadeh

**Affiliations:** 1. Department of Physiotherapy, School of Primary Health Care, Medicine, Nursing and Health Sciences, Monash University, Melbourne, Australia.; 2. Department of Medicine at Royal Melbourne Hospital, University of Melbourne, Melbourne, Australia.; 3. Monash Alfred Psychiatry Research Centre, Alfred and Monash University Central Clinical School, Melbourne, Australia.

**Keywords:** Transcranial magnetic stimulation, Reliability, Evoked response variability, First dorsal interosseous muscles

## Abstract

**Introduction:**

Transcranial magnetic stimulation (TMS) is a useful tool for assessment of corticospinal excitability (CSE) changes in both healthy individuals and patients with brain disorders. The usefulness of TMS-elicited motor evoked potentials (MEPs) for the assessment of CSE in a clinical context depends on their intra-and inter-session reliability. This study aimed to evaluate if removal of initial MEPs elicited by using two types of TMS techniques influences the reliability scores and whether this effect is different in blocks with variable number of MEPs.

**Methods:**

Twenty-three healthy participants were recruited in this study. The stimulus intensity was set at 120% of resting motor threshold (RMT) for one group while the stimulus intensity was adjusted to record MEPs up to 1 mV for the other group. Twenty MEPs were recorded at 3 time points on 2 separate days. An intra-class correlation coefficient (ICC) reliability with absolute agreement and analysis of variance model were used to assess reliability of the MEP amplitudes for blocks with variable number of MEPs.

**Results:**

A decrease in ICC values was observed with removal of 3 or 5 MEPs in both techniques when compared to all MEP responses in any given block. Therefore, removal of the first 3 or 5 MEPs failed to further increase the reliability of MEP responses.

**Conclusion:**

Our findings revealed that a greater number of trials involving averaged MEPs can influence TMS reliability more than removal of the first trials.

## 1. Introduction

Transcranial magnetic stimulation (TMS) is a useful tool for assessment of corticospinal excitability (CSE) changes in both healthy individuals and patients with brain disorders (Barker et al., 1987; Rossini et al., 1994; Liepert et al., 2000). The magnetic pulses induced by TMS over the contralateral primary motor cortex (M1) can pass through the scalp and induce a response known as “motor evoked potential” (MEP) in the target muscle. This response is recorded using surface electromyography (EMG) electrodes placed over the muscle of interest (Malcolm et al., 2006).The peak-to-peak amplitude of the elicited MEPs is an indication of changing CSE. Smaller amplitudes indicate lower excitability, while larger amplitudes suggest higher CSE (Chipchase et al., 2012).

Literature review indicates that there is a high degree of variability in the TMS-induced resting MEPs (Kiers et al., 1993; Ellaway et al., 1998).This variability could result from technical factors such as orientation, location, and stability of the TMS coil (Barker et al., 1987; Hill et al., 2000; Chipchase et al., 2012). However, variability in MEP responses remains even after controlling these factors. This inherent variability could result from neurophysiological changes in the CSE pathway (Truccolo et al., 2002). More variability might be expected in the amplitude of the first few MEPs due to changes in regional cerebral flow (Mochizuki et al., 2006) and changes in excitatory synaptic drive to corticospinal neurons (Ellaway et al., 1998).

The first few MEP responses might be larger than the subsequent MEPs (Brasil-Neto et al., 1994), and the increased variability in initial MEPs can affect TMS reliability (Schmidt et al., 2009).Therefore, removal of the first, more fluctuating MEPs might increase the averaged reliability scores. In TMS studies, CSE could be assessed using 2 different techniques. In the first technique, the test stimulus is calculated as a ratio of a resting motor threshold (RMT) such as 120% RMT. In the second technique, the test stimulus is adjusted to produce MEP responses up to 1 mV, which is commonly used in paired-pulse TMS studies. Since there is an inverse relationship between variability of MEP responses and TMS stimulus intensity (Kiers et al., 1993), the MEPs evoked by the 1 mV technique are less subject to variability, which may be less affected by more variable and fluctuating initial MEPs.

The literature suggests that increasing the number of evoked MEPs increases the TMS reliability (Ellaway et al., 1998; Truccolo et al., 2002; Kamen, 2004; Bastani & Jaberzadeh, 2012). Little is known about how removal of the first few MEPs affects the reliability scores of TMS techniques. In this study, we investigate the effects of removal of the initial elicited MEPs on reliability scores, and also whether this effect is different in blocks with different MEP numbers. We hypothesised that removal of three or five initial MEPs should increase reliability. We also hypothesised that the removal of the initial MEPs should have more profound effects on enhancement of reliability than the number of MEPs in each block.

## 2. Methods

### 2.1. Participants

Eighteen healthy participants were recruited in this-study and divided into two groups to assess the reliability of MEPs responses induced by two types of TMS techniques. Nine participants (8 females and one male with the mean [SD] age of 27[11.6] y) were included in one group where the test stimulus was considered at 120% RMT. In the other group (9 females, with the mean [SD] age of 23.5[2.8] y), the test stimulus was adjusted at 1 mV. Handedness of the participants was assessed using the Edinburgh Handedness Questionnaire (Oldfield, 1971). The dominant hand was tested in each participant. Of 18 participants, 16 were right-hand dominant. Participants were screened for contraindication to TMS applications. They provided their written informed consent prior to the experiments. All protocols used were approved by the Human Research Ethics Committees at Monash University and conformed to the Declaration of Helsinki.

### 2.2. Measurements

#### 2.2.1. Electromyography

Participants were tested in a sitting position with forearm supported in a pronated position. A standard skin preparation (Gilmore & Meyers, 1983) procedure was performed for each electrode placement site. EMG electrodes were placed on the first dorsal interosseous (FDI) muscle of the dominant hand with an inter-electrode distance of 2 cm. A ground electrode was placed ipsi-laterally over the styloid process of the ulna bone. All EMG signals were filtered, amplified (10 Hz–500 Hz × 1000), and sampled at 1000 Hz. All data were recorded on a PC via a commercially available software (Chart™ software, ADInstrument, Australia) and a laboratory analogue-digital interface (The Power Lab 8/30, ADInstrument, Australia) for later off-line analysis.

#### 2.2.2. Motor evoked potentials

Single pulse magnetic stimuli were delivered using two stimulators with a figure-of-eight coil. A Magstim 2002 (Magstim Company Limited, UK) stimulator was used for recording MEPs with intensity of 120% RMT in group 1, and a MagPro R30 (MagOption) stimulator (MagVenture Denmark) was used for recording MEPs using the second technique in group 2. In both groups, the coil was placed over the dominant M1, i.e. contralateral to the muscle of interest. The orientation of the coil was set at an angle of 45° to the midline and tangential to the scalp. In this orientation, the induced current flow is directed from posterior to anterior. The coil was moved around the M1 of the FDI muscle to determine the optimal site of stimulation. After localizing this site, known as a hot spot, the coil position was marked on the scalp as a reference. Coil position and orientation were constantly assessed throughout the experiment to minimize technical inconsistencies.

After localizing the hot spot, RMT was measured. RMT is defined as the lowest intensity to induce at least 5 MEPs larger than 50 μV in peak-to-peak amplitude out of 10 consecutive stimuli to find RMT, also the intensity of the stimulator was decreased in steps of 2% of the maximum stimulator output. The test stimulus was set at 120% of each individual’s RMTs in group 1 and adjusted up to produce MEP responses of about 1 mV in group 2.

### 2.3. Procedure

Each participant was tested in two separate testing sessions. The first session involved two sets of data collection. FDI muscle MEPs were recorded before and immediately after a 20-minute break in which subjects were recommended to do activities such as reading books or magazines. During each testing session, 20 MEPs with interpulse intervals of 10 seconds (Vaseghi et al., 2015) were recorded. A follow-up session was held at least 72 hours after the first session. All participants were assessed at the same time of day in both sessions to avoid diurnal variations.

### 2.4. Data analysis

In both groups, 20 stimuli were delivered, with 10 seconds interstimulus interval. The averaged MEPs at each time point were calculated for the first 10 (Block 1), first 15 (Block 2), and all 20 MEPs (Block 3). Then the averaged MEPs were also calculated after removal of the first 3 and the first 5 MEPs in each block. The effects of removal of the first 3 and the first 5 MEPs in each block were evaluated using intraclass correlation coefficients (ICCs) with absolute agreement and a 2-way mixed model. Repeated measures analysis of variance (ANOVA) was used to detect any differences between the averaged MEPs across 3 time points at any given block.

SPSS (version 20) was used for the data analysis. A significance level of P<0.05 was adopted for all conditions. Post hoc tests (Student t test with Bonferroni correction) were performed where indicated.

## 3. Results

A total of 18 individuals were recruited for this study. Three subjects took part in both groups while the rest of the subjects participated in only one group. In group 1 (n=13), stimulus intensity was delivered at 120% RMT. In group 2 (n=13), the average stimulus intensity required to produce MEPs of about 1 mV was 139% RMT (with Min and Max 104% and 185 % RMT). The average (SD) handedness scores were 79.4(25.2) and 86.7(9.8) in groups 1 and 2, respectively.

In Table 1, the results of the ICCs and F tests values in all blocks with different number of trials are shown for group 1. The ICC values ranged from 0.75 to 0.92 in blocks 1, 2, and 3 indicating that increasing the number of trials can lead to an increase in ICC values. The results of ICCs in all blocks with removal of the first 3 or 5 MEPs revealed slightly decreased reliability for the FDI MEP responses. More reduction in ICC values was observed with removing the first 3 MEPs in all blocks, compared to removal of the first 5 MEPs (Table 1). No differences were observed in the averages of MEP sizes in blocks with different number of trials between any time points across two sessions. As shown in Table 2, similar results were observed in group 2 with test intensity of up to 1 mV. The range of the ICCs in this group was lower than that in group 1, but similar pattern was found in the results of the ICC values. ICCs in all blocks with removal of the first 3 or 5 MEPs revealed slightly decreased reliability for the FDI MEP responses. More reduction in ICCs was obtained with removing the first 3 MEPs in all blocks, compared to removal of the first 5 MEPs (Table 2). There were no significant differences in the average MEP size at any time points in any given block (Table 2).

**Table 1:** The results of ICCs and F test in three blocks 10, 15, and 20 MEPs in three types of conditions (all trials, after removal of the first three or five MEPs) at three time points across the two sessions (MEPs 120% RMT).

**Test Intensity=120% RMT N=13**	**T1 Session 1 (Mean±SD)**	**T2 Session 1 (Mean±SD)**	**T1 Session 2 (Mean±SD)**	**F (2, 24)**	**P**	**ICCs**	**P**
Block 1a (1–10 MEPs)	0.78±0.47	0.70±0.66	0.68±0.50	0.325	0.726	0.851	0.000
Block 1b (4–10 MEPs)	0.71±0.39	0.65±0.69	0.63±0.45	0.197	0.864	0.754	0.002
Block 1c (6–10 MEPs)	0.71±0.43	0.64±0.68	0.68±0.49	0.134	0.875	0.830	0.000
Block 2a (1–15 MEPs)	0.74±0.41	0.71±0.64	0.70±0.41	0.069	0.934	0.897	0.000
Block 2b (4–15 MEPs)	0.69±0.35	0.69±0.66	0.67±0.36	0.022	0.978	0.839	0.000
Block 2c (6–15 MEPs)	0.68±0.37	0.69±0.66	0.70± 0.37	0.009	0.991	0.881	0.000
Block 3a (1–20 MEPs)	0.72±0.40	0.77±0.65	0.69±0.40	0.397	0.677	0.922	0.000
Block 3b (4–20 MEPs)	0.67±0.35	0.76±0.67	0.68±0.37	0.514	0.605	0.893	0.000
Block 3c (6–20 MEPs)	0.67±0.38	0.77±0.67	0.69±0.38	0.521	0.601	0.895	0.000

Significant results are bold.

**Table 2:** The results of ICCs and f tests in three blocks 10, 15 and 20 MEPs in three types of conditions (all trials, after removal of the first three or five MEPs) at three time points across the two sessions (MEPs∼1 mV).

**Stimulus Intensity=MEPs 1mV N=13**	**T1 Session 1 (Mean±SD)**	**T2 Session 1 (Mean±SD)**	**T1 Session 2 (Mean±SD)**	**F (2, 24)**	**P**	**ICCs**	**P**
Block 1a (1–10 MEPs)	1.09±0.24	1.12±0.35	1.01±0.19	0.791	0.465	0.533	0.056
Block 1b (4–10 MEPs)	1.05±0.37	1.102±0.39	1.05±0.28	0.094	0.911	0.422	0.135
Block 1c (6–10 MEPs)	1±0.42	1.17± 0.48	1.07±0.42	0.699	0.510	0.564	0.043
Block 2a (1–15 MEPs)	1.06±0.20	1.07±0.32	1.05±0.25	0.073	0.930	0.721	0.005
Block 2b (4–15 MEPs)	1.03±0.34	1.03±0.38	1.1±0.32	0.267	0.768	0.609	0.029
Block 2c (6–15 MEPs)	0.99±0.30	1.08±0.38	1.09±0.36	0.527	0.597	0.694	0.008
Block 3a (1–20 MEPs)	1.03±0.21	1.06±0.29	1.04±0.25	0.082	0.94	0.770	0.002
Block 3b (4–20 MEPs)	1.02±0.31	1.02±0.34	1.1±0.30	4.37	0.651	0.684	0.009
Block 3c (6–20 MEPs)	0.98±0.303	1.06±0.33	1.07±0.33	0.56	0.578	0.733	0.003

The ICC values for this group ranges from 0.42 to 0.77 in blocks 1, 2, and 3 indicating that raising the number of trials can lead to an increase in ICC values.

**Figure 1:**
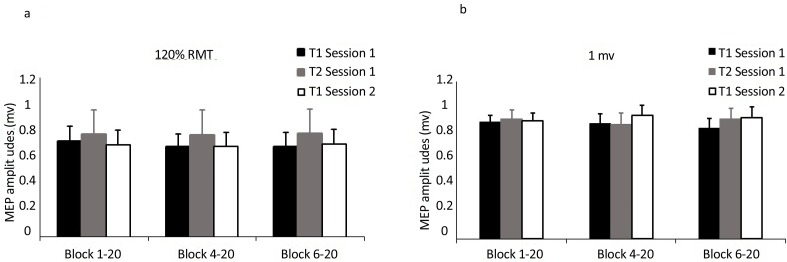
Comparison of MEPs amplitude in blocks of 20 MEPs in 3 conditions (all trials, after removal of the first 3 and 5 MEPs at 3 time points across two sessions. a) group 120% RMT, b) group 1 mV.

Figure 1 shows the results of comparison of MEPs amplitude in block 20 MEP responses in 3 conditions (all trials, after removal of the first 3 and 5 MEPs) for two types of TMS methods, 120% RMT and intensity to elicit 1 mV MEPs.

## 4. Discussion

In this study, we assessed the reliability of TMS induced MEP, using two types of TMS techniques (120% RMT and 1 mV), and considering removal of data for the first few trials in each block. The hypothesis that the removal of initial MEPs would increase the MEP reliability was refuted by the results. Our results have shown that reliability scores decrease with removal of the first 3 or 5 MEPs in each block, except for block 10 at 1 mV intensity in which removing the first 5 trials slightly increased ICCs compared to all 10 MEPs. In both techniques, we observed more reduction in ICC values with removing the first 3 MEPs in all blocks, compared to removal of the first 5 MEPs. The results also indicate that, compared to removal of the first few MEPs, the number of MEPs in each block has a more profound effect on the enhancement of reliability in both techniques.

The patterns of variability of MEP size and the mechanisms responsible for this variability have not been completely determined. Changes in the level of synchrony of neuronal pulse activity and spontaneous changes in motor neuron excitability are often identified as the sources of such variability (Scriven & Paul, 1987; Kriz et al., 1995; Tallon-Baudry et al., 1996, 1997; Tallon-Baudry et al., 1998; Srinivasan et al., 1999; Hunter et al., 2009; Sankarasubramanian et al., 2015; Viviani and Lacquaniti, 2015). Large changes in CSE might result in greater fluctuations in MEP amplitude during the first few trials of TMS (Brasil-Neto et al., 1994; Ellaway et al., 1998), which can affect overall reliability of elicited MEPs. However, our finding demonstrated that removal of the first few trials resulted in lower values of MEP reliability when compared to removing all trials in any given block. The ICC values recorded for all three blocks of 10, 15, and 20 MEPs showed a rise in reliability score with increasing the number of trials, which is in agreement with those results suggesting that there is a relationship between the number of trials and reliability score (Kiers et al., 1993; Kamen, 2004; Christie et al., 2007; Bastani & Jaberzadeh, 2012).

In the current study, different impacts on reliability scores are achieved by removing the first 3 or 5 MEPs. Different values of ICCs in a given block with removal of the first 3 or 5 trials indicated that not only the number of MEPs, but also the number of removed initial trials can influence reliability of this response. In the current study, a slight increase in ICC values was observed in blocks with the first 5 trials removed, compared to exclusion of the first 3 trials. This finding can be explained by the increased homogeneity in MEP amplitudes being expected after the first 5 MEPs (Maher et al., 2003), which is line with some studies that reported ICC values above 0.6 for blocks of 5 MEPs (Kamen, 2004; Christie et al., 2007; Bastani and Jaberzadeh, 2012).

Similar patterns in reliability scores were found between two types of TMS techniques. The only difference was found in block 10 MEPs using TMS technique 1 mV. In this case, by removing the first 5 trials, ICCs slightly increased. In addition, there is a clear trend that, after removal of first few trials, the SD of MEP increased in the 1 mV technique more than that in the 120% RMT technique. This increase was larger for the removal of 5 trials than the removal of 3 trials, indicating the first 3 or 5 MEPs were very close to the mean value of all trials.

Taken together, to receive reliable responses, increasing the number of trials might be more effective than removing the first few trials. Therefore, using 20 MEPs allows us to accurately measure mean MEP amplitude as a valid outcome. More studies are needed to find out factors which contribute to MEP variability and the reliability of MEP responses.

There are some limitations in this research. Healthy young participants were assessed in this study, therefore, our results cannot be extrapolated to other populations such as patients or elderly people. Furthermore, the intensity of the stimuli was set at 120% of RMT or 1 mV at rest condition, therefore the findings could not be generalized to other TMS intensities and active conditions. Future studies must be conducted on patients, on other age ranges, and for active and rest conditions at different TMS intensities.

This study demonstrated that a greater number of trials involving averaged MEPs can influence TMS reliability more than removal of the first few trials in a given block. On the other hand, removal of more variable and fluctuating initial MEPs did not have a significant impact on overall reliability of TMS-induced MEPs between two techniques (1 mV and 120% RMT).
